# College Dispensary—Clinical Lectures before the Class of Rush Medical College

**Published:** 1847-04

**Authors:** J. R. Bradway

**Affiliations:** A Member of the Class; Chicago


ARTICLE IV.

### COLLEGE DISPENSARY.

### *Clinical Lectures before the Class of Rush Medical College.*

[Reported for the Journal by J. R. BRADWAY, M. D., a member of the class.]

The Lectures given upon the different cases which presented themselves at the Dispensary were published in the first and second volumes of this Journal, but were discontinued during the last year on account of the similarity of the cases to those before described. Lately, however, since the adoption of the charity by the public authorities of the county, it has acquired such an extension in its usefulness as to furnish, at all times, new and useful cases for clinical instruction. The lectures given on these will hereafter be regularly reported for this Journal.

CASE I.—*January* 20, 1847. F. D., aged 22 years, a German, of strong robust form, and bilious temperament, had been affected with *jaundice* for about a week, without suffering ill health previous to the attack. After the commencement of the discoloration of the skin, the patient was, for two or three days, troubled with a diarrhœa, with light colored discharges, which, at this time, had ceased. Urine exceedingly high colored, sclerotic coat of the eyes of a saffron hue, lucid cornea tinged and the skin of a deep yellow. Complains of no pain, but suffers from nausea almost constantly, especially when in the erect position. Discoloration had increased regularly and rapidly since the onset of the disease.

*Remarks* by Prof. EVANS.—GENTLEMEN : You have here a well marked case of *Jaundice.* It is rather a symptom of certain diseased conditions of the biliary organs, than a disease itself. The pathological condition is this: In health, the bile is eliminated from the blood for the double purpose of purifying the circulating mass and of exerting its peculiar influence on the bowels. When it is not separated and thrown off by the liver, either the bile, as such, or the constituent principles of which it is composed, are retained abnormally in the system, and hence the discoloration. And it is said to exert a narcotic influence, to which is attributed the languor experienced by patients suffering with jaundice. The absence of the natural stimulus furnished to the mucous membrane o the intestines causes irritation, and diarrhœa, with light colored fæces, showing absence of bile, as we have it in the case before us.

Jaundice depends upon an impaired secretion of bile, or an obstruction in its elimination.

There are numerous forms of disease that give rise to jaundice ; and you must be extremely careful, here, not to prescribe for the *name*, but for the pathological condition producing the symptoms.

Jaundice may depend upon an organic lesion of the liver, either acute or chronic. In the case before us we have no tenderness over the region in which the liver is situated, which is the best diagnostic symptom of acute disease of the organ. The attack is sudden, with previous good health, and thus the history of the case proves it not to be a chronic lesion of structure. Nor have we the pain in the region of the scapula usually attendant upon structural lesions of the liver; nor yet have we the tumefaction of the liver. It may depend upon inflammation of the duodenum, causing a swelling of the coats of that intestine at the valvular opening of the ductus communis choledochus, so as to obstruct the flow of the bile. But in this case, the symptoms of duodenitis are not present.

It may depend upon disease of the heart. Dr. Graves thinks disease of the liver frequently is the result of disease of the heart. But here the action of the heart is natural, and the circulation is regular. If it were not, we might look out for congestion of the liver as a cause of the jaundice. Congestion of the portal circulation and, consequently, of the liver is a very common cause of functional derangement of this organ in the western country. Here, where disease is so universally a succession of paroxysms of congestion and of re-action, as in the chill and fever, it is not strange that jaundice should be common from functional derangements of the liver. It is not reasonable to expect that the liver should, during these congestions, continue to perform its function healthily. The extreme vascularity of the organ—being endowed with three distinct sets of blood-vessels — those from the portal circle, the hepatic arteries, and the hepatic veins, all of which are without valves to prevent regurgitation—subjects it, more than any other important organ, to suffer from lesions in the circulation. But, in this case, if it depend upon congestion, it must be entirely local, as the patient has been free from any general derangement of the circulation.

Jaundice may depend upon a tumor or abscess being so situated as to compress the biliary or common duct; but this comes on gradually, generally in case of tumors; and the symptoms of the formation of abscess have not been present.

A loaded condition of the duodenum making pressure upon the valvular opening of the common bile duct into that intestine, may give rise to jaundice; but this would give us a tumor in the region of the duodenum, which is not here.

It may depend upon an unhealthy secretion giving rise to obstructions in consequence of the presence, in the ducts, of gall stones, or viscid bile. This is the form of jaundice that is most rapid and sudden in its onset. The presence of gall stones, where they form obstructions to the flow of bile, gives great pain, resembling colic, generally followed by vomiting, which, from its relaxing influence, and the mechanical impetus given the retained bile, often effects relief. But the other form, which we have in the case before us, is attended with no very painful symptom. Nausea, which is very distressing, generally attends it, as in this case, and sometimes a fit of vomiting gives relief as in case of gall stones.

The probabilities of cure in cases of jaundice, are determined by the nature of the affection upon which the case is dependent. In this case we make a favorable prognosis.

The indications of cure are, to remove obstructions, restore the healthy action of the liver, and give tone to the general system.

By the administration of an emetic of tart. ant. et pot., combined with ipecac., we may, to a certain extent, fill two of these indications—to remove the viscid bile from the duct, and alter and increase the secretion. After the operation of the emetic, the secretion may be promoted by the administration of five grain doses of calomel at intervals of four hours until three are taken, combined with Dover's powder to prevent it from passing off too rapidly by the bowels; this to be followed by castor oil.

*January* 22. The patient returned, much relieved from the sickness of the stomach, the most distressing symptom.

Calomel and Dover's powder repeated, to be followed by castor oil, as before. Patient directed to use tincture of the bark of the root of Wild Cherry, (*Prunus Virginiana*,) half a wine-glassful three times daily.

*January* 25. Patient much improved. Directed to continue the use of the tincture. Under this treatment he was speedily restored to health.

Case II—*Surgical Clinic.*—A. B., aged 32, of Will county, Illinois, applied January 1, 1847, for relief from a dropsical affection, and also on account of a fistulæ of the anus under which he had been laboring for eight years. The serous effusion was removed by the use of the decoction of the bark of wahoo—a new remedy, with the properties of which the readers of the Journal are already acquainted, and whose good effects have often been conspicuous during the past winter in the treatment of effusions following upon fevers.

Prof. Brainard, upon commencing the treatment of the fistula, remarked, that "it is a disease which very often exists for many years, either without the patient being aware of its presence, or without giving rise to sufficient inconvenience to induce him to apply for relief. The disease consists of a narrow passage existing at the side of the rectum, having in some cases, both an internal and an external opening; in others an internal, and in others still an external opening only.

The first is called complete; the second incomplete internal; and the last incomplete external fistula. Fistula results from abscesses which, forming in the vicinity of the anus, pass into this state from the movements of the sphincter ani, the contractions of which prevent it from healing. Or it may commence in the mucous ducts or follicles which are situated in the depression between the internal and external sphincters. It is not unfrequently found in connection with phthisis pulmonalis, or other diseases producing or following upon an impaired constitution. In these cases, the fistulous passage often extends around the rectum, or for a considerable distance upward at its side, passing sometimes within the tuburosity of the ischium, and having several openings externally.

The existence of a fistula of the anus may be suspected from pain and heat of the part, attended, usually, by a moisture produced by a slight but constant discharge of pus from its orifice. This orifice, when external, is often concealed by folds of skin, but its locality will generally be indicated by a small mucous tubercle.

For the purpose of examination, the patient should be placed in a convenient position, and a probe passed into the fistula. The finger, well oiled, should at this time be passed into the rectum, by which means the extent and direction of the fistula can be ascertained.

In the present instance, we find it to be very extensive; the external opening being in the perineum, and the internal one two inches from the anus.

The treatment of fistula of the anus divides itself into two different classes of means; the one having for its object the cure without, the second by means of, an operation. A recent fistula may often be cured by means of laxatives which prevent the injury of the parts by hardened fæces, whilst irritation is excited by nit. arg., sulph. cupri, or other escharotics. In old cases, and especially those which are extensive, this mode is entirely ineffectual, and it becomes necessary, either to resort to some operation or leave the case to nature.

There are instances in which the latter method is preferable. They are all those where the disease is connected with phthisis or other form of cachexia, when any operation would not only be useless, but might be injurious. The case before us partakes somewhat of this character, the patient being of broken constitution and but recently relieved, as you know, from a severe dropsical affection. Desirous of doing nothing which might give too severe a shock to the constitution, we shall, in this case not perform the usual operation for fistula, which consists in dividing, upon a director, all that portion of tissue situated between it and the rectum, and which, when they are widely separated, is of considerable severity.

Desirous, however, of doing something for his relief, we shall adopt a middle course, and use a means little known and used in the profession, but which, in a few cases, will be found to possess advantages over any other. The method of treatment alluded to is that by ligature. A piece of common saddler's silk should be passed, by means of an eyed probe, through the fistula, and brought out at the anus, so as to embrace all that portion of tissue usually divided in operating; the two extremities of the ligature are then to be joined together by means of a slip knot, so as to allow of its being easily drawn tight. This is to be immediately closed and repeated daily until the parts embraced are diminished gradually by ulceration, which requires several weeks, but which is attended by but little pain."

The operation of introducing the ligature was then performed as above described, and at the present time, March 6th, but a small portion of tissue remains to be divided. The cure might have been long since completed but for the want of care on the part of the patient in tightening the ligature. His general health is improved, and he will soon be capable of performing the ordinary labor of a man.

Case. III—*Phymosis.*—J. B., aged, 14 years, from Kendall county Illinois, came to the Dispensary February 20th, 1847, for relief from a phymosis. On examination, the orifice of the prepuce was found to be so small as barely to admit the extremity of a common sized probe. The father of the boy stated that the malformation had existed from birth, but had been several times operated upon by simple division without success. Prof. Brainard stated, that "before performing the operation required in this case, it was desirable to consider, for a short time, the causes producing the difficulty and the reasons requiring an operation. The prepuce in young persons is usually so contracted before the glans penis as to render its retraction difficult or impossible, and this state often continues during life without producing more than a simple inconvenience, and not by any means requiring an operation for its relief. At times, however, from the accumulation of vitiated secretion or the deposition of venereal virus beneath the prepuce, ulcerations take place difficult to heal and requiring incisions for their full exposure.

In other cases the occurrence of irritation at the orifice attended with slight ulceration produces such a contraction as to render the escape of the urine slow and difficult. Such is the case in the instance before us. The contraction in this case has, however, apparently been produced by the operations which were attempted for his relief; for, in each case, the contraction was carried to a greater extent than before. This tendency of the various canals to close under the influence of any irritation, which is often witnessed in the passages of the external ear, the mouth, the vagina, the urethra, etc., should fix the attention of surgeons, since, in many cases, by producing it artificially, we have a means of closing various preternatural openings, such as vesico-vaginal fistula, cleft palate, etc. But in order to obtain its full effect, we must imitate the action of disease itself, and keep up the irritation constantly for many months, and even years. There is scarcely any orifice or canal, either natural or otherwise, which can resist such continued applications without contracting and eventually closing altogether. It should also teach us that simply dilating, cauterizing or enlarging the orifice with the knife is a temporary means, followed, for the most part, by aggravation of the deformity. The operation which will be found most effectual in cases of phymosis consists either in dividing the prepuce freely from its extremity to the base of the glans penis and stitching together the external and internal coverings, or in simply circumcising the patient and stitching the two layers of integuments together all round at their points of division. In the present case the latter operation is performed in consequence of the redundancy of the skin of the part."

The operation was then performed, and at the end of two weeks the patient returned home, cured, except at one or two small points not yet entirely cicatrized.

Case IV—*Hydrocele of the Cord and Tunica Vaginalis in a child eighteen months old.*—This child was brought to the Dispensary February 26th, 1847, and presented a case remarkable for its size and the rapidity of its development. Prof. Brainard remarked that he should not go into a description of all the diagnostic signs of this affection, since the sensation of fluctuation was so distinct as to leave no doubt as to the fluid character of the contents of the tumor, and its translucency clearly reveals its serous nature. Neither should he describe all the different methods formerly employed for its cure. At present scarcely any other operation for hydrocele is thought of except injection; and the fluid used is generally a solution of iodine and hyd. potass., in various proportions, according to the degree of strength required. The peculiarity of this operation as practiced by Prof. Brainard consists in his using a very fine trochar, not larger than a middle sized knitting needle; and having allowed the fluid to flow out through the canala, injecting two fluidrachms of the above named solution, *which is all allowed to remain.* By this means sufficient inflammation to secure adhesion is produced without danger of suppuration.

The operation was, in this case, performed with success.

Case V—*Partial Closure of the Mouth resulting from Mercurial Salivation.*—A. Duffy, aged 8 years, came to the Dispensary with this affection, of one year's standing. The cicatrix was on the left side, extending between the gums of the bi-cuspid teeth, narrow, and sufficiently long to allow of the mouth being opened half an inch. Prof. Brainard explained in full the causes and character of this kind of deformity, which he stated to be extremely common in the western States. For this description we refer our readers to the Am. Journ. of the Med. Sci., for August, 1843, where may be found, also, a figure of the instrument used by Dr. B. for separating the jaws in difficult cases. The present case being comparatively slight, the cicatrix was simply divided by a horizontal cut, which allowed the jaws fully to open. When thus open, the anterior and posterior margins of the wound were approximated by stitches so that only a vertical line was left. This simple operation was invented for this case, and was fully successful; the boy enjoying, at the present time, full movement of the lower jaw.

*Chicago, March 9th*, 1847.

